# Mechanisms of Reduced Susceptibility to Cefiderocol Among Isolates from the CREDIBLE-CR and APEKS-NP Clinical Trials

**DOI:** 10.1089/mdr.2021.0180

**Published:** 2022-04-18

**Authors:** Patrice Nordmann, Ryan K. Shields, Yohei Doi, Miki Takemura, Roger Echols, Yuko Matsunaga, Yoshinori Yamano

**Affiliations:** ^1^ Medical and Molecular Microbiology, Department of Medicine, Faculty of Science and Medicine, University of Fribourg, Fribourg, Switzerland.; ^2^ Division of Infectious Diseases, University of Pittsburgh, Pittsburgh, Pennsylvania, USA.; ^3^ Pharmaceutical Research Division, Shionogi & Co., Ltd., Osaka, Japan.; ^4^ Infectious Disease Drug Development Consulting, LLC, Easton, Connecticut, USA.; ^5^ Shionogi, Inc., Florham Park, New Jersey, USA.

**Keywords:** cefiderocol, MIC increase, mutation, resistance, susceptibility, whole-genome sequencing

## Abstract

The objective of this study was to characterize isolates with reduced susceptibility to cefiderocol in patients receiving cefiderocol for nosocomial pneumonia or carbapenem-resistant infections in the Phase 3 APEKS-NP and CREDIBLE-CR studies. Susceptibility testing of isolates was conducted at a central laboratory, and post-treatment changes were evaluated according to available breakpoints for cefiderocol. Whole-genome sequencing and multilocus sequence typing were performed for isolates to confirm their origin and identify mutations. Five (APEKS-NP) and nine (CREDIBLE-CR) isolates demonstrated a ≥ 4-fold minimum inhibitory concentration (MIC) increase compared with genetically related baseline isolates; most remained susceptible to cefiderocol despite the ≥4-fold MIC increase. Mutations in β-lactamases or penicillin-binding protein (PBP) were identified in 4/14 isolates: one *Enterobacter cloacae* (amino acid [AA] substitution [A313P] in ACT-17); two *Acinetobacter baumannii* (one PBP3 AA substitution [H370Y], one with OXA-23 substitutions [N85I and P225S]); and one *Pseudomonas aeruginosa* (PDC-30 [4AA deletion “TPMA” position 316–319]). Cloning experiments using isogenic *Escherichia coli* strains containing wild-type and those mutant cephalosporinase enzymes show that the mutant enzymes may contribute to decreased susceptibility to cefiderocol. Pharmacokinetic data were available for nine patients, for whom cefiderocol exposures exceeded 100% *f*T > 4 × MIC_._ No clear pattern between mutations and development or extent of MIC increases was observed. No mutations were identified in genes related to iron transport, including *fiu*, *cirA*, *piuA/C*, and *pirA*, among recovered Gram-negative isolates. Clinicaltrials.gov: APEKS-NP: NCT03032380; CREDIBLE-CR: NCT02714595.

## Introduction

Cefiderocol is a novel siderophore cephalosporin with broad-spectrum activity against aerobic Gram-negative bacteria, including Enterobacterales and the nonfermenters *Acinetobacter baumannii*, *Pseudomonas aeruginosa*, and *Stenotrophomonas maltophilia*.^1,2^ Cefiderocol is an iron chelator, which enters bacteria by active uptake through iron transport channels,^3–5^ and is the first approved siderophore cephalosporin.^6,7^ It was developed to target carbapenem-resistant (CR) isolates, irrespective of the resistance mechanism.^1,8^ Cefiderocol is stable against hydrolysis by enzymes belonging to Ambler Classes A, B, C, and D, and is active against isolates with porin channel mutations or upregulated efflux pumps.^1,9^

As with any new antibiotic, the development of reduced susceptibility is inevitable with clinical use.^10–13^ Monitoring such changes during clinical use plays a crucial role in detecting and understanding the global spread of resistance and identifying strategies to mitigate the emergence of resistance.

Cefiderocol was investigated in two randomized, prospective, controlled Phase 3 studies (APEKS-NP in patients with nosocomial pneumonia [NP]^14^ and CREDIBLE-CR in patients with CR infections^15^) that reported similar clinical and microbiological outcomes between cefiderocol and comparator agents. All-cause mortality (ACM) rates at Day 14, 28, and end of study were comparable between cefiderocol and high-dose, extended-infusion meropenem in APEKS-NP.^14^

In CREDIBLE-CR, infections caused by *Acinetobacter* spp. (mainly pneumonia and bloodstream infection [BSI]/sepsis) resulted in higher rates of ACM with cefiderocol than with the best available therapy (BAT) at each time point.^15^ The difference in mortality was explained partially by imbalances between treatment arms in the baseline clinical characteristics of patients with *Acinetobacter* spp. infections, including a greater incidence of moderate or severe renal dysfunction, intensive care unit stay at randomization, ongoing septic shock at screening or within 31 days before randomization in the cefiderocol compared with the BAT arm.^15^ The median treatment duration was similar between treatment arms in both studies.^14,15^

In both studies, isolates with increases in post-treatment minimum inhibitory concentrations (MICs) of ≥4-fold were noted for all treatments, and occurred in similar proportions of patients in the cefiderocol and comparator arms (4.8% and 3.9% of patients, respectively, in APEKS-NP; 15.0% and 13.2% of patients, respectively, in CREDIBLE-CR).^14,15^

The objective of the current investigation was to further characterize isolates from the APEKS-NP and CREDIBLE-CR studies that demonstrated reduced susceptibility, defined as a ≥ 4-fold baseline to post-treatment increase in the cefiderocol MIC.

## Materials and Methods

### Studies and susceptibility testing

APEKS-NP (NCT03032380) was a 1:1 randomized, double-blind, multicenter, noninferiority Phase 3 study in patients with NP, comparing treatment with cefiderocol (2 g, q8h, 3-hour infusion) or high-dose, extended-infusion meropenem (2 g, q8h, 3-hour infusion), for 7–14 days (or up to 21 days at the discretion of the investigator). While no adjunctive Gram-negative therapy was allowed, patients in both arms received at least 5 days of linezolid treatment for Gram-positive coverage. Exclusion criteria included pneumonia caused by a CR pathogen known at the time of randomization.^14^

CREDIBLE-CR (NCT02714595) was a 2:1 randomized, open-label, multicenter, descriptive Phase 3 study in critically ill patients with serious infections (NP, BSI/sepsis, complicated urinary tract infections [cUTIs]) caused by CR Gram-negative pathogens. Patients received cefiderocol (2 g, q8h, 3-hour infusion) or BAT (≤3 agents against Gram-negative bacteria, with dosing based on local practice) for 7–14 days (or up to 21 days at the discretion of the investigator). Exclusion criteria included receipt of potentially effective antibiotics for the current CR infection within 72 hours before randomization (with a continuous duration of >24 hours for cUTI or >36 hours for other infections).^15^

In both studies, appropriate microbiological samples were collected at screening, early assessment (EA, Days 3–4), end of treatment (EOT, last day of treatment), and test of cure (TOC, EOT +7 days) to evaluate microbiological outcomes. Culture and susceptibility testing were carried out locally, and specimens were frozen and transferred to the central laboratory for species confirmation, antibiotic susceptibility, and testing for the presence of extended-spectrum β-lactamases and/or carbapenemases (IHMA, Schaumburg, IL).^14,15^

All isolates with reduced susceptibility (defined as a ≥ 4-fold MIC increase between baseline and post-treatment assessments) underwent repeat susceptibility testing at the central laboratory by broth microdilution in iron-depleted media in duplicate. Median MIC values were used to assess MIC fold changes. MIC susceptibility results were assessed according to the Clinical and Laboratory Standards Institute (CLSI), U.S. Food and Drug Administration (FDA), and European Committee on Antimicrobial Susceptibility Testing (EUCAST) breakpoints.^16–18^

In both studies, blood sampling at steady state was performed in most patients to assess cefiderocol pharmacokinetics (PK).^14,15^ Free drug level in plasma, based on the previously determined *in vitro* unbound fraction of 0.422,^19^ was used to calculate the percentage of time unbound drug concentrations were above the MIC (%*f*T>MIC) and the more stringent 4 × MIC (%*f*T > 4 × MIC) with reference to baseline MIC values.

Both studies received institutional review board/independent ethics committee approval.^14,15^

### Molecular characterization of genes of interest

Whole-genome sequencing (WGS) was performed for isolates with confirmed ≥4-fold cefiderocol MIC increase from baseline, and multilocus sequence typing (MLST) was used to confirm their origin. To determine MLST type, the best matching genome from GenBank was identified for each genome and used to guide assembly. The appropriate MLST scheme was selected for the genome identified, and the allelic profile determined using the CLC Genomics Workbench, version 12 (QIAGEN, Inc., Germantown, MD). Only isolates found to belong to the same sequence type (ST) pre- and post-treatment underwent investigation for gene mutations potentially related to cefiderocol resistance.

In brief, WGS analysis involved pelleting cells from 3 mL liquid cultures grown overnight from one colony in Brain Heart Infusion broth (Sigma-Aldrich, St. Louis, MO) at 37°C with shaking. DNA was extracted using the DNeasy Ultraclean Microbial extraction kit (QIAGEN, Inc.). Sequencing, involving libraries prepared from an Illumina Nextera XT DNA Library Preparation Kit (Illumina, Inc., San Diego, CA), was performed by Illumina HiSeq (Illumina, Inc.), using 2 × 150 bp paired end reads with a target coverage depth of 100 × . All analyses were conducted using the CLC Genomics Workbench, version 12. *De novo* assemblies of each genome were queried for the analysis of the previously known resistance-related genes using the Center for Genomic Epidemiology database for resistance genes (ResFinder).^20^

All known β-lactamase genes available in ResFinder database^20^ were investigated for mutations. To better detect highly diverse AmpC genes, for which few variants have been characterized, thresholds for minimum nucleotide sequence identity and minimum sequence length were set to 72% and 80%, respectively. Results that were <100% identical or did not contain the full-length sequence were appended as such for clarity.

Other genes of interest were selected based on *in vitro* findings and based on the mode of action of cefiderocol (Table 1), and reference sequences (Supplementary Tables S1 and S2) were used to search (tBLASTn) *de novo* assemblies for each genome. Positive hits were assessed for the presence of changes that introduced premature stop codons in the coding sequence (“truncated” vs. “no truncation” if these lesions were not identified).

**Table 1.** Specific Genes Investigated with Whole-Genome Sequencing for the Presence of *De Novo* Mutations Using tBLASTn Search

Species	Enterobacterales	Pseudomonas aeruginosa	Acinetobacter baumannii	Stenotrophomonas maltophilia
β-lactamases^a^	All	All	All	All
Iron transporter	*fiu, cirA*	*piuA, piuC, pirA*	*piuC, bauA, pfeA, feoB, feoA*	*piuA, piuC, pirA*
Iron transport related	*exbB, exbD, tonB*	*exbB, exbD, tonB*	*exbB, exbD, tonB*	*exbB, exbD, tonB*
Others	*ftsI* (PBP3) *BaeS/R, OmpR/EnvZ* (two-component regulation) *pcnB* (polynucleotide adenyl-transferase)	*ftsI* (PBP3) *pvdS* (pyoverdine synthesis regulator)	*ftsI* (PBP3)	*ftsI* (PBP2)
Porin	*ompC, ompF*	*oprD*	*carO*	N/A

^a^ All known β-lactamases available in ResFinder gene database.^20^

N/A, not applicable.

Investigation for any nonsynonymous deletions or insertions within coding regions was performed to detect potential amino acid (AA) changes between pre- and post-treatment isolates in genes that may be the source of acquired resistance.

For penicillin-binding protein (PBP) gene identification, reference sequences for the protein products of *ftsI* were BLAST searched on a species-specific basis. tBLASTn was used to find the gene with the lowest *E* value (database) to the reference sequence (query), for which mutations encoding AA changes were identified. AA substitutions compared with the wild-type reference sequences are reported.

For TonB-dependent siderophore uptake receptor genes and porin genes, reference sequences and predetermined porin genes and their homologs, respectively, (Supplementary Tables S1 and S2) were used to search (tBLASTn) *de novo* assemblies for each genome.

The position of the AA residue, which was confirmed to be mutated between baseline and post-treatment, was shown from the N-terminus without excluding the signal sequences.

### Construction of recombinant isogenic *Escherichia coli* strains

*E. coli* isogenic strains expressing wild-type and mutant β-lactamases, which were identified in the post-treatment isolates showing reduced susceptibility to cefiderocol, were constructed to observe the effect of β-lactamase gene mutations on cefiderocol susceptibility. Amplification of DNA fragments of the β-lactamase genes was performed from the first codon to the stop codon by polymerase chain reaction, and the fragments were introduced into the multicloning site in pET9a (Invitrogen™, Carlsbad, CA). The constructed plasmid was introduced into *E. coli* BL21 (DE3) (Invitrogen), and the susceptibility of these recombinant strains to cefiderocol was determined in triplicate by broth microdilution in iron-depleted media in the presence of 1 mM of isopropyl β-d-1-thiogalactopyranoside (IPTG) to induce the expression of β-lactamases.

## Results

### APEKS-NP

In the APEKS-NP study, reduced susceptibility of isolates, which were genetically related (*i.e.*, with the same MLST and/or core genome single nucleotide polymorphisms) at baseline and post-treatment visits, was reported for seven isolates from six patients receiving cefiderocol (4.8% of patients [6/124 patients in the modified intent-to-treat population with confirmed Gram-negative pathogen at baseline]). One of these isolates was *Serratia marcescens* collected from a patient coinfected with *Enterobacter cloacae* that also showed reduced susceptibility. There was no MLST scheme for *S. marcescens*, but based on the nucleotide sequence of the genes and the susceptibility profile to other antibiotics, the post-treatment isolate was considered to have emerged from the pretreatment isolate.

Initial susceptibility testing performed in APEKS-NP revealed 4- to 8-fold post-treatment increases in cefiderocol MICs against all seven isolates, which remained ≤4 μg/mL. Upon repeat susceptibility testing, only five of the seven isolates demonstrated reduced susceptibility; the remaining two isolates showed minimal MIC changes and were excluded from further analysis: *Klebsiella pneumoniae* (median MIC: baseline ≤0.03 μg/mL, EOT 0.03 μg/mL) and *Klebsiella aerogenes* (median MIC: baseline 0.125 μg/mL, TOC 0.25 μg/mL). Only one (*E. cloacae*) demonstrated a post-treatment MIC >4 μg/mL (Table 2).

**Table 2.** Pre- and Post-Cefiderocol MIC Changes, Resistance Criteria, Identified β-Lactamases, and Post-Treatment Whole-Genome Sequencing-Identified Mutations in Multilocus Sequence Typing-Confirmed Isogenic Isolates with *a* ≥ 4-Fold Post-Treatment Increase in MIC: Data from APEKS-NP

				Cefiderocol MIC retests				Resistance according to established criteria based on median MIC						
Species and site of infection	Time point for MIC increase	Original MIC (μg/mL)	Fold of baseline MIC	MIC #2 (μg/mL)	MIC #3 (μg/mL)	Median MIC (μg/mL)	Fold of median baseline MIC	CLSI S/I/R ≤4/8/≥16	FDA S/I/R ≤4/8/≥16 ENT & ≤1/2/≥4 NF	EUCAST S/R ≤2/>2 (ENT, PA)	fT >4 × MIC (%)	C_min_(μg/mL)	β-Lactamase identified at baseline and post-treatment (WGS)	Post-treatment mutation identified (WGS)
*Klebsiella aerogenes* Lung (VAP)	Baseline	0.06		0.06	0.12	0.06		S	S	S	100	15.9	AmpC^a^	
	EA	0.5	8 ×	0.25	0.25	0.25	4 ×	S	S	S			AmpC^a^	Not identified
*Klebsiella pneumoniae* Lung (HCAP)	Baseline	0.06		≤0.03	0.03	0.03		S	S	S	100	7.68	SHV-1	
	EA	0.25	4 ×	0.25	0.5	0.25	8 ×	S	S	S			SHV-1	Not identified
*K. pneumoniae* Lung (HAP)	Baseline	0.25		0.25	0.5	0.25		S	S	S	100	13.4	CTX-M-15; OXA-1-like; TEM-1; SHV-11	
	TOC	1	4 ×	1	1	1	4 ×	S	S	S			CTX-M-15; OXA-1-like; SHV-11	Not identified
***Enterobacter cloacae***^b^ Lung (VAP)	**Baseline**	**1**		**2**	**4**	**2**		**S**	**S**	**S**	**NA**	**NA**	**ACT-17^c^**	
	**EOT**	**4**	**4 ×**	**8**	**8**	**8**	**4 ×**	**I**	**I**	**R**			**ACT-17-like^c^**	**ACT-17-like (A313P)**
*Serratia marcescens*^b^ Lung (VAP)	Baseline	0.06		0.12	0.25	0.12		S	S	S	NA	NA	SRT-2-like^d^	
	EA	0.25	4 ×	0.5	1	0.5	4 ×	S	S	S			SRT-2-like^d^	Not identified

Bold text refers to isolates with mutations.

^a^ 100% match to CMY/MIR/ACT/EC family class C β-lactamase (Accession #: WP_110884515); variant name is unidentified.^33^

^b^ The *E. cloacae* and *S. marcescens* isolates were found in the same patient in the same biospecimen. This enzyme belongs to Class C AmpC enzymes.

^c^ This enzyme belongs to Class C AmpC enzymes in *Enterobacter* spp.

^d^ This enzyme belongs to Class C AmpC enzymes in *Serratia* spp.

CLSI, Clinical and Laboratory Standards Institute; *C*_min_, minimum free concentration at steady state; EA, early assessment; ENT, Enterobacterales; EOT, end of treatment; EUCAST, European Committee on Antimicrobial Susceptibility Testing; FDA, U.S. Food and Drug Administration; %fT >4 × MIC, % fraction of time of unbound drug above 4 × MIC; HAP, hospital-acquired pneumonia; HCAP, healthcare-associated pneumonia; I, intermediate; MIC, minimum inhibitory concentration; NA, not applicable; NF, nonfermenters; PA, *Pseudomonas aeruginosa*; R, resistant; S, susceptible; TOC, test of cure; VAP, ventilator-associated pneumonia; WGS, whole-genome sequencing.

According to WGS, all five isolates had β-lactamase genes present at baseline (Table 2). An AA substitution, (A313P) in ACT-17 (a naturally occurring class C β-lactamase), was identified in an *E. cloacae* isolate with pre- and post-treatment median MICs of 2 and 8 μg/mL (4-fold increase), respectively (Table 2). The introduction of A313P mutation in ACT-17 led to a 2-fold increase in the cefiderocol MIC in isogenic *E. coli* strains (Table 3). No other mutations in β-lactamase genes were identified.

**Table 3.** Cefiderocol MIC Changes after Insertion of β-lactamase Gene Mutations into *Escherichia coli* Isogenic Strains Observed in Isolates Showing Reduced Susceptibility to Cefiderocol

Test strains	Cefiderocol MIC (μg/mL)
BL21(DE3)/pET9a (vector control)	0.125
BL21(DE3)/pET9a::ACT-17	0.125
BL21(DE3)/pET9a::ACT-17-like (A313P)	0.25
BL21(DE3)/pET9a::OXA-23	0.125
BL21(DE3)/pET9a::OXA-23-like (N85I, P225S)	0.125
BL21(DE3)/pET9a::PDC-30	0.125
BL21(DE3)/pET9a::PDC-30-like (T316-A319del)	1

ACT-17 mutation was observed in one *Enterobacter cloacae* isolate in APEKS-NP. OXA-23 mutation was observed in one *Acinetobacter baumannii* isolate in CREDIBLE-CR. PDC-30 mutation was observed in one *Pseudomonas aeruginosa* isolate in CREDIBLE-CR.

PK data were available for three of four patients from whom serial isolates demonstrated reduced susceptibility. For all three patients, cefiderocol concentrations achieved 100% *f*T > 4 × MIC (Table 2). Minimum plasma concentrations at steady state (*C*_min_) ranged from 7.68 to 15.9 μg/mL.

### CREDIBLE-CR

In the CREDIBLE-CR study, reduced susceptibility of isolates, which were genetically related at baseline and post-treatment visits, was identified in 11 isolates from 11 patients receiving cefiderocol (13.8% of patients [11/80 patients in the CR microbiological intent-to-treat population with ≥1 confirmed CR pathogen at baseline]). According to initial susceptibility testing, cefiderocol MICs remained ≤4 μg/mL for 8 of the 11 isolates. One isolate showed a 16-fold MIC increase (*A. baumannii*), and one had a 128-fold MIC increase (*P. aeruginosa*).

After repeat susceptibility testing, two isolates were excluded from further analysis: one *A. baumannii* (median MIC: baseline 0.5 μg/mL, EA 1 μg/mL) and one *K. pneumoniae* (median MIC: baseline 0.25 μg/mL, TOC 0.25 μg/mL). The magnitude of increase in median MIC for the other nine isolates ranged from 4- to 128-fold and remained ≤4 μg/mL for six isolates (Table 4). Eight of the nine isolates were from patients with NP, and the remaining isolate was from a patient with cUTI (*P. aeruginosa*). Three patients received combination treatment (Table 4).

**Table 4.** Pre- and Post-Cefiderocol MIC Changes, Resistance Criteria, Identified β-Lactamases, and Post-Treatment Whole-Genome Sequencing-Identified Mutations in Multilocus Sequence Typing-Confirmed Isogenic Isolates with *a* ≥ 4-Fold Post-Treatment Increase in MIC: Data from CREDIBLE-CR

				Cefiderocol MIC retests				Resistance according to established criteria, based on median MIC						
Species and site of infection	Time point for MIC increase	Original MIC (μg/mL)	Fold of baseline MIC	MIC #2 (μg/mL)	MIC #3 (μg/mL)	Median MIC (μg/mL)	Fold of median baseline MIC	CLSI S/I/R ≤4/8/≥16	FDA S/I/R ≤4/8/≥16 ENT & ≤1/2/≥4 NF	EUCAST S/R ≤2/>2 (ENT, PA)	fT >4 × MIC (%)	C_min_ (μg/mL)	β-Lactamase identified at baseline and post-treatment (WGS)	Post-treatment mutation identified (WGS)
***Acinetobacter baumannii*** Lung (VAP)	**Baseline**	**0.25**		**1**	**1**	**1**		**S**	**S**	**S**	**NA**	**NA**	**ADC-25-like^d^; OXA-23; OXA-66^d^; TEM-1D**	
	**Day 14**	**4**	**16 ×**	**2**	**4**	**4**	**4 ×**	**S**	**R**	**R**			**ADC-25-like^d^; OXA-23; OXA-66^d^; TEM-1D**	**PBP3 (H370Y)**
***A. baumannii*** Lung (VAP)	**Baseline**	**1**		**1**	**2**	**1**		**S**	**S**	**S**	**100**	**26.4**	**ADC-25-like^d^; OXA-23; OXA-69^d^**	
	**EOT**	**8**	**8 ×**	**64**	**64**	**64**	**64 ×**	**R**	**R**	**R**			**ADC-25-like^d^; OXA-23-like; OXA-69^d^**	**OXA-23 (N85I and P225S)**
*A. baumannii* Lung (VAP)^a^	Baseline	1		0.25	1	1		S	S	S	100	26.7	ADC-25-like^**d**^; OXA-23; OXA-71^**d**^	
	Unscheduled (Day 10)	8	8 ×	8	8	8	8 ×	I	R	R			ADC-25-like^**d**^; OXA-23; OXA-71^**d**^	Not identified
*K. pneumoniae* Lung (VAP)	Baseline	0.06		≤0.03	0.06	0.06		S	S	S	100	8.24	SHV-61-like^e^	
	Unscheduled (Day 8)	0.5	8 ×	1	1	1	16 ×	S	S	S			SHV-61-like^e^ partial CDS; TEM-18	Not identified
*K. pneumoniae* Lung (HAP)^b^	Baseline	0.25		0.12	0.25	0.25		S	S	S	NA	NA	KPC-2; SHV-1	
	Day 14	2	8 ×	4	2	2	8 ×	S	S	S			KPC-2; SHV-1	Not identified
*Pseudomonas aeruginosa* Urine (cUTI)	Baseline	0.12		0.12	0.12	0.12		S	S	S	100	7.3	OXA-847^f^; PDC-11; VIM-2	
	EA	16	128 ×	16	16	16	128 ×	R	R	R			OXA-847^f^; PDC-11; VIM-2	Not identified
***P. aeruginosa*** **Lung (HAP)**	**EA**	**0.25**		**0.12**	**0.12**	**0.12**		**S**	**S**	**S**	**NA**	**NA**	**OXA-488^f^; PDC-30**	
	**EOT**	**2**	**8 ×**	**2**	**2**	**2**	**16 ×**	**S**	**I**	**S**			**OXA-488^f^; PDC-30-like**	**PDC30 (4 AA deletion “TPMA” position 316–319)**
*P. aeruginosa* Lung (VAP)	Baseline	0.5		1	0.25	0.5		S	S	S	100	15.2	OXA-50-like^g^; PDC-11	
	EOT	2	4 ×	4	2	2	4 ×	S	I	S			OXA-50-like^g^; PDC-11	Not identified
*Stenotrophomonas maltophilia* Lung (VAP)^c^	Baseline	0.06		0.06	0.12	0.06		NA	NA	NA	100	51.7	L1, L2^h^	
	EOT	0.25	4 ×	0.12	0.25	0.25	4 ×	NA	NA	NA			L1, L2^h^	Not identified

Bold text refers to isolates with mutations.

^a^ Treatment with combination of cefiderocol+ampicillin-sulbactam.

^b^ Treatment with combination of cefiderocol+tigecycline.

^c^ Treatment with combination of cefiderocol+levofloxacin.

^d^ In *A. baumannii*, OXA-66, OXA-69, OXA-71, and ADC-25 are naturally occurring and weakly expressed.

^e^ In *K. pneumoniae*, SHV-61 is naturally occurring.

^f^ In *P. aeruginosa*, OXA-847 and OXA-488 are naturally occurring and weakly expressed, and do not interfere with common resistance patterns. They belong to OXA-50-like group of β-lactamases.

^g^ OXA-50–like groups of β-lactamases are naturally occurring enzymes and weakly expressed.

^h^ L1 and L2 are naturally occurring β-lactamases; L1: a metallo-β-lactamase and L2: an extended-spectrum β-lactamase, respectively. L1 was not available in the ResFinder database but was detected by manual analysis of the assembled sequence.

CDS, coding sequences; cUTI, complicated urinary tract infection; NT, not tested.

According to WGS, targeted mutations were identified in three of the nine isolates with reduced susceptibility (Table 4). In one *A. baumannii* isolate, a PBP3 AA substitution (H370Y) was noted, with median pre- and post-treatment MICs of 1 and 4 μg/mL, respectively (4-fold increase). Two substitutions (N85I and P225S) in an acquired carbapenemase OXA-23 were identified in another *A. baumannii* isolate, with median pre- and post-treatment MICs of 1 μg/mL and 64 μg/mL, respectively (64-fold increase).

The third isolate was a *P. aeruginosa* with a mutation in PDC-30 [4 AA deletion “TPMA” position 316–319] and median pre- and post-treatment MICs of 0.12 and 2 μg/mL, respectively (16-fold increase). The introduction of OXA-23 N85I and P225S mutations into isogenic *E. coli* strains did not change cefiderocol MIC. However, introduction of PDC-30 “TPMA” 316–319 deletion caused an 8-fold increase in cefiderocol MIC (Table 3). The *P. aeruginosa* isolate, collected from a cUTI patient, with a 128-fold MIC increase according to median susceptibility testing (pretreatment 0.12 μg/mL, post-treatment 16 μg/mL) had no known mutations in the genes evaluated.

PK data were available for six of the nine patients infected by isolates with confirmed reduced susceptibility. In all six cases, cefiderocol exposures of 100% *f*T > 4 × MIC were achieved. *C*_min_ values ranged from 7.3 to 51.7 μg/mL (Table 4).

In both studies, no nonsynonymous mutations in genes related to efflux pumps or transcriptional regulators of iron transporters were identified, including *fiu*, *cirA*, *piuA/C,* and *pirA* (Table 1).

## Discussion

In this investigation, we identified 14 isolates with confirmed reduced susceptibility after treatment with cefiderocol in the APEKS-NP and CREDIBLE-CR studies. Thirteen of the 14 isolates were from patients with NP. Not surprisingly, these types of isolates were more common in patients from the CREDIBLE-CR study than in those from the APEKS-NP study (11.3% vs. 3.2%, respectively). This frequency mirrors that of isolates with ≥4-fold MIC increases from patients in the comparator arms of each study based on initial MIC data (13% vs. 4%).^14,15^

The pathogens in the CREDIBLE-CR study were carbapenem resistant and frequently multidrug resistant with few treatment options,^15^ while 78% of the pathogens in the APEKS-NP study were carbapenem susceptible.^14^ The difference in the underlying susceptibility pattern may account partially for the difference in the proportion of isolates with ≥4-fold post-treatment MIC increases.

Most of the isolates developing median ≥4-fold cefiderocol MIC increases remained susceptible according to CLSI/FDA/EUCAST guidelines. Only two isolates, *P. aeruginosa* (128-fold MIC increase) and *A. baumannii* (64-fold MIC increase), both from CREDIBLE-CR, were classified as phenotypically resistant according to all three criteria.

Mutations were identified in four isolates. One *E. cloacae* was resistant according to EUCAST, but intermediate according to the CLSI/FDA criteria, one *A. baumannii* was susceptible by CLSI but resistant by the EUCAST/FDA criteria, one *A. baumannii* was resistant according to all three criteria, and one *P. aeruginosa* was susceptible by CLSI/EUCAST and intermediate by the FDA criteria. Among these four isolates, results with isogenic *E. coli* strains suggest that the PDC-30 mutation was the likely cause of the cefiderocol MIC increase in *P. aeruginosa*.

The ACT-17 mutation might be related to the reduced cefiderocol susceptibility of *E. cloacae*, but this is unclear due to relative change in MIC. On balance, OXA-23 mutations at positions N85I and P225S did not alter the cefiderocol MIC in an isogenic *E. coli* background. Given the infrequency of target site mutations, we were unable to explain reduced susceptibility in most post-treatment isolates. There was also no pattern between type of emergent mutation and MIC fold increase.

Importantly, no mutations were identified in genes previously shown in other clinical isolates and nonclinical studies to be associated with cefiderocol MIC increases, including those involved in iron transport.^21–23^ Chemostat models demonstrated that *K. pneumoniae* mutants that have shown cefiderocol MIC increases did not appear with humanized cefiderocol exposures.^23^ However, one study of *A. baumannii* clinical isolates suggested the potential involvement of PBP3 mutation in cefiderocol resistance.^22^ It is possible that mutations in unknown/uninvestigated genes may have played a role in the changes in cefiderocol MICs.

In APEKS-NP, cefiderocol was administered as monotherapy by design.^14^ In CREDIBLE-CR, 83% of patients received cefiderocol monotherapy, although one adjunctive agent could be administered to patients with NP or BSI/sepsis.^15^ In the current investigation, three of nine patients received cefiderocol in combination therapy (Table 3), suggesting that the addition of a second agent against Gram-negative bacteria did not necessarily mitigate the emergence of resistance. PK blood sampling at steady state showed that in all cases tested, cefiderocol plasma concentrations achieved a pharmacodynamic target of 100% *f*T > 4 × MIC.

A recent population PK analysis suggested that there was no association between cefiderocol exposure and clinical or microbiological efficacy outcomes; cefiderocol plasma concentrations were high, and 100% *f*T>MIC was achieved in 97% of patients in each of the APEKS-NP and CREDIBLE-CR studies.^24^ Plasma drug exposures were similar across infection sites.^24^

According to a recent Phase 1b study in pneumonia patients requiring mechanical ventilation and standard-of-care antibiotics, cefiderocol concentration in the epithelial lining fluid (ELF) was higher at 2 hours after the end of infusion compared with that measured at the end of infusion, suggesting that the clearance of cefiderocol is slower from the lung than from plasma.^25^ The ELF concentrations also suggested that cefiderocol penetration into ELF is adequate for pathogens with MIC values of ≤4 μg/mL.^25^ It is worth mentioning that in the current investigation, 10 of the 13 respiratory isolates had a median post-treatment MIC of ≤4 μg/mL despite *a* ≥ 4-fold increase in the cefiderocol MIC.

The clinical significance of MIC increases varies, and increases do not always translate into clinical resistance. There was no correlation between outcomes and observed MIC increases in the isolates from APEKS-NP and CREDIBLE-CR.^14,26^ In a recent case report on the compassionate use of cefiderocol for 21 days, a tracheal aspirate sample obtained 10 days after the EOT revealed two morphological variants of an extensively drug-resistant *P. aeruginosa* isolate, both of which were associated with MIC increases (from 0.25 to 0.5 μg/mL and 1 μg/mL, respectively)^27^; however, both remained susceptible to cefiderocol treatment according to CLSI criteria.

Understanding and monitoring the mechanisms responsible for development of resistance to cefiderocol will be essential in providing clinical solutions on how to treat problematic infections, as well as to devising strategies to prevent the development of resistance. Previous studies have noted an issue with *P. aeruginosa* adaptive resistance (regrowth of bacteria observed in the presence of an antibiotic) to previous siderophore-conjugated antibiotics.^4^ In studies with SMC-3176 and MB-1, *in vivo* efficacy was lower than that predicted by *in vitro* activity for some *P. aeruginosa* isolates.^28,29^

In the case of MB-1, the effects were linked to the endogenous siderophore pyoverdine and its uptake by PiuA or PirA, and resistance was reversed in the presence of an efflux pump inhibitor.^29,30^ Adaptive resistance to *P. aeruginosa* has not been observed with cefiderocol. *In vivo* studies investigating MB-1, SMC-3176, and cefiderocol in the murine thigh infection model confirmed the variable efficacy of MB-1 and SMC-3176, and showed that cefiderocol exhibited potent *in vivo* activity and sustained efficacy.^31^

The limitations of the current investigations are that susceptibility for isolates with ≥4-fold post-treatment MIC increases in the comparator arms was not retested and their post-treatment MICs were not confirmed; therefore, comparison of the proportion of these types of isolates could only be made based on original results.

In addition, there was no investigation of potential heteroresistance, which has been recently reported,^32^ but the clinical significance of this phenomenon has not been established. Although no increase in cefiderocol MIC was seen with the expression of mutant OXA-23 enzyme in the isogenic *E. coli* cloning experiments, the enzyme may not have been fully functional or fully expressed on this genetic background. It is also a possibility that genes not investigated in this study may contribute to the reduced susceptibility.

## Conclusion

We identified 14 isolates from 13 patients in the APEKS-NP and CREDIBLE-CR studies with confirmed ≥4-fold increases in cefiderocol MICs after cefiderocol treatment. Mutations were uncommon, and 64% of isolates remained susceptible to cefiderocol according to the CLSI interpretative criteria. No consistency was found across isolates in terms of emerging mutations, and no mutations were identified in genes related to iron transport in Gram-negative bacteria.

## Supplementary Material

Supplemental data

Supplemental data
