# Supplementary material for: Mechanisms of Reduced Susceptibility to Cefiderocol Among Isolates from the CREDIBLE-CR and APEKS-NP Clinical Trials
Source: Microb Drug Resist. 2022 Apr 18;28(4):398–407. doi: 10.1089/mdr.2021.0180 (PMC9058874; doi:10.1089/mdr.2021.0180)
Supplement: Supplemental data [file Suppl_TableS1.docx]

**SUPPLEMENTARY TABLES**

Supplementary Table 1. Reference Sequences for Genes of Interest: APEKS-NP

| *Organism* | *Gene* | *Genome accession* | *Locus tag/CDS* | *Protein accession* |
| --- | --- | --- | --- | --- |
| *Citrobacter freundii* | *ftsI* | NZ_CP026677.1 | AM350_RS03015 | WP_003829538.1 |
| *Citrobacter freundii* | *ompC-like* | JMTA01000004.1 | GCFR_00645 | KFB99940.1 |
| *Citrobacter freundii* | *ompF-like* | JMTA01000013.1 | GCFR_01674 | KFB98469.1 |
| *Citrobacter freundii* | *pirA-like (fepA)* | NZ_CP026238.1 | C2U43_RS06820 | WP_103284856.1 |
| *Enterobacter cloacae* | *cirA-like* | NZ_LPQI01000052.1 | ASU78_15685 | WP_058678819.1 |
| *Enterobacter cloacae* | *ftsI* | CP001918.1 | ECL_01481 | ADF61041.1 |
| *Enterobacter cloacae* | *ompC-like* | CP001918.1 | ECL_03519 | ADF63053 |
| *Enterobacter cloacae* | *ompF-like* | NC_014121.1 | ECL_02724 | YP_003613214.1 |
| *Enterobacter cloacae* | *baeS* | NC_04121 | ECL_03405 | YP_003613888.1 |
| *Enterobacter cloacae* | *envZ* | NC_04121 | ECL_04768 | YP_003615244.1 |
| *Enterobacter cloacae* | *exbB* | NC_04121 | ECL_04329 | YP_003614808.1 |
| *Enterobacter cloacae* | *exbD* | NC_04121 | ECL_04328 | YP_003614807.1 |
| *Enterobacter cloacae* | *pcnB* | NC_04121 | ECL_00947 | YP_003611459.1 |
| *Enterobacter cloacae* | *tonB* | NC_04121 | ECL_01647 | YP_003612154.1 |
| *Klebsiella aerogenes* | *cirA* | NZ_QVMZ01000004.1 | D0N43_RS09290 | WP_015706114.1 |
| *Klebsiella aerogenes* | *ftsI* | NZ_QVMZ01000004.1 | D0N43_RS07885 | WP_015705932.1 |
| *Klebsiella aerogenes* | *ompC-like* | NZ_QVMZ01000004.1 | D0N43_RS09530 | WP_015706141.1 |
| *Klebsiella aerogenes* | *ompF-like* | NZ_QVMZ01000025.1 | D0N43_RS23570 | WP_015367453.1 |
| *Klebsiella aerogenes* | *baeS* | NZ_QVMZ01 000000 | D0N43_RS08 925 | WP_015706068. 1 |
| *Klebsiella aerogenes* | *envZ* | NZ_QVMZ01 000000 | D0N43_RS11 245 | WP_015369373. 1 |
| *Klebsiella aerogenes* | *exbB* | NZ_QVMZ01 000000 | D0N43_RS06 605 | WP_015369670. 1 |
| *Klebsiella aerogenes* | *exbD* | NZ_QVMZ01 000000 | D0N43_RS06 600 | WP_015369671. 1 |
| *Klebsiella aerogenes* | *pcnB* | NZ_QVMZ01 000000 | D0N43_RS22 415 | WP_026612395. 1 |
| *Klebsiella aerogenes* | *tonB* | NZ_QVMZ01 000000 | D0N43_RS12 330 | WP_015705108. 1 |
| *Klebsiella pneumoniae* | *baeS* | NZ_KN04681 8 | DR88_RS050 15 | WP_004149058. 1 |
| *Klebsiella pneumoniae* | *cirA* | NZ_KN04681 8 | DR88_RS053 30 | WP_032443975. 1 |
| *Klebsiella pneumoniae* | *envZ* | NZ_KN04681 8 | DR88_RS040 50 | WP_002920333. 1 |
| *Klebsiella pneumoniae* | *exbB* | NZ_KN04681 8 | DR88_RS096 80 | WP_004174395. 1 |
| *Klebsiella pneumoniae* | *exbD* | NZ_KN04681 8 | DR88_RS096 75 | WP_002916785. 1 |
| *Klebsiella pneumoniae* | *fiu* | NZ_KN04681 8 | DR88_RS161 50 | WP_023316750. 1 |
| *Klebsiella pneumoniae* | *ompK35 (ompF-like)* | NZ_KN04681 8 | DR88_RS177 00 | WP_004141771. 1 |
| *Klebsiella pneumoniae* | *ompK36 (ompC-like)* | NZ_KN04681 8 | DR88_RS055 45 | WP_004149145. 1 |
| *Klebsiella pneumoniae* | *pbp3* | NZ_KN04681 8 | DR88_RS221 30 | WP_002888559. 1 |
| *Klebsiella pneumoniae* | *pcnB* | CP027146 | CSC00_1301 | AVJ86299.1^a^ |
| *Klebsiella pneumoniae* | *tonB* | NZ_KN04681 8 | DR88_RS123 90 | WP_025861279. 1 |
| *Serratia marcescens* | *ftsI* | KN050642.1 | DP21_3084 | KFL03598.1 |
| *Serratia marcescens* | *ompC-like* | KN050642.1 | DP21_2139 | KFL01276.1 |
| *Serratia marcescens* | *ompF-like* | NZ_CP018924.1 | BVG88_05495 | WP_017892288.1 |
| *Serratia marcescens* | *pirA-like (fepA)* | NZ_CM008894.1 | CS367_RS17065 | WP_033636747.1 |
| *Serratia marcescens* | *baeS* | NZ_CP01892 4 | BVG88_RS15 730 | WP_033644061. 1 |
| *Serratia marcescens* | *envZ* | NZ_CP01892 4 | BVG88_RS20 595 | WP_033645286. 1 |
| *Serratia marcescens* | *exbB* | NZ_CP01892 4 | BVG88_RS18 730 | WP_080273417. 1 |
| *Serratia marcescens* | *exbD* | NZ_CP01892 4 | BVG88_RS18 725 | WP_004937307. 1 |
| *Serratia marcescens* | *pcnB* | NZ_CP01892 4 | BVG88_RS17 895 | WP_071883579. 1 |
| *Serratia marcescens* | *tonB* | NZ_CP01892 4 | BVG88_RS10 685 | WP_033643281. 1 |
| *Acinetobacter baumannii* | *carO* | KP658473.1 | 1..750 | AKL79738.1 |
| *Acinetobacter baumannii* | *ftsI* | CP000521.1 | A1S_3204 | ABO13597.2 |
| *Acinetobacter baumannii* | *oprD* | NZ_QCXW010 00025.1 | DJ41_RS08835 | WP_000910009.1 |
| *Acinetobacter baumannii* | *pirA-like* | NZ_PXUK0100 0006.1 | C6Y25_RS0316 0 | WP_000044170.1 |
| *Acinetobacter baumannii* | *piuA-like* | NZ_VNWQ010 00118.1 | FQK03_RS160 75 | WP_005135841.1 |
| *Acinetobacter baumannii* | *carO* | KP658473.1 | 1..750 | AKL79738.1 |
| *Acinetobacter baumannii* | *ftsI* | CP000521.1 | A1S_3204 | ABO13597.2 |
| *Acinetobacter baumannii* | *oprD* | NZ_QCXW01000025.1 | DJ41_RS08835 | WP_000910009.1 |
| *Acinetobacter baumannii* | *pirA-like* | NZ_PXUK01000006.1 | C6Y25_RS03160 | WP_000044170.1 |
| *Acinetobacter baumannii* | *piuA-like* | NZ_VNWQ01000118.1 | FQK03_RS16075 | WP_005135841.1 |
| *Pseudomonas aeruginosa* | *ftsI* | AE004091.2 | PA4418 | AAG07806.1 |
| *Pseudomonas aeruginosa* | *oprD* | NC_002516.2 | PA0958 | NP_249649.1 |
| *Pseudomonas aeruginosa* | *pirA* | AE004091.2 | PA0931 | AAG04320.1 |
| *Pseudomonas aeruginosa* | *piuA* | AF051690.1 | 2133..4394 | AAC06215.1 |
| *Pseudomonas aeruginosa* | *piuD* | CP027166.1 | CSB94_2935 | AVK00042.1 |

^a^Alternative to ATCC 13883 since gene in that strain is truncated
