# Supplementary material for: Mechanisms of Reduced Susceptibility to Cefiderocol Among Isolates from the CREDIBLE-CR and APEKS-NP Clinical Trials
Source: Microb Drug Resist. 2022 Apr 18;28(4):398–407. doi: 10.1089/mdr.2021.0180 (PMC9058874; doi:10.1089/mdr.2021.0180)
Supplement: Supplemental data [file Suppl_TableS2.docx]

Supplementary Table 2. Reference Sequences for Genes of Interest: CREDIBLE-CR

| *Organism* | *Gene* | *Genome accession* | *Locus tag* | *Protein accession* |
| --- | --- | --- | --- | --- |
| *Escherichia coli* | *fiu* | NC_000913 | b2155 | NP_416660.1 |
| *Escherichia coli* | *cir* | NC_000913 | b0805 | NP_415326.1 |
| *Escherichia coli* | *envZ* | NC_000913 | b3404 | NP_417863.1 |
| *Escherichia coli* | *baeS* | NC_000913 | b2078 | NP_416582.1 |
| *Escherichia coli* | *tonB* | NC_000913 | b1252 | NP_415768.1 |
| *Escherichia coli* | *pcnB* | NC_000913 | b0143 | NP_414685.4 |
| *Escherichia coli* | *exbB* | NC_000913 | b3006 | NP_417479.1 |
| *Escherichia coli* | *exbD* | NC_000913 | b3005 | NP_417478.1 |
| *Klebsiella pneumoniae* | *fiu* | NZ_KN04681 8 | DR88_RS16150 | WP_023316750.1 |
| *Klebsiella pneumoniae* | *cir* | NZ_KN04681 8 | DR88_RS05330 | WP_032443975.1 |
| *Klebsiella pneumoniae* | *envZ* | NZ_KN04681 8 | DR88_RS04050 | WP_002920333.1 |
| *Klebsiella pneumoniae* | *baeS* | NZ_KN04681 8 | DR88_RS05015 | WP_004149058.1 |
| *Klebsiella pneumoniae* | *tonB* | NZ_KN04681 8 | DR88_RS12390 | WP_025861279.1 |
| *Klebsiella pneumoniae* | *pcnB* | CP027146 | CSC00_1301 | AVJ86299.1 |
| *Klebsiella pneumoniae* | *exbB* | NZ_KN04681 8 | DR88_RS09680 | WP_004174395.1 |
| *Klebsiella pneumoniae* | *exbD* | NZ_KN04681 8 | DR88_RS09675 | WP_002916785.1 |
| *Acinetobacter baumannii* | *exbB 1* | NZ_CP00925 7 | IX87_RS02265 | WP_024437313.1 |
| *Acinetobacter baumannii* | *exbB 2* | NZ_CP00925 7 | IX87_RS16540 | WP_001011664.1 |
| *Acinetobacter baumannii* | *exbB 3* | NZ_CP00925 7 | IX87_RS18495 | WP_031971902.1 |
| *Acinetobacter baumannii* | *exbD 1* | NZ_CP00925 7 | IX87_RS02260 | WP_000669684.1 |
| *Acinetobacter baumannii* | *exbD 2* | NZ_CP00925 7 | IX87_RS16545 | WP_000885431.1 |
| *Acinetobacter baumannii* | *exbD 3* | NZ_CP00925 7 | IX87_RS18490 | WP_000525862.1 |
| *Acinetobacter baumannii* | *exbD 4* | NZ_CP00925 7 | IX87_RS18485 | WP_000525666.1 |
| *Acinetobacter baumannii* | *tonB1* | NZ_CP00925 7 | IX87_RS16535 | WP_000730960.1 |
| *Acinetobacter baumannii* | *tonB2* | NZ_CP00925 7 | IX87_RS11595 | WP_024437247.1 |
| *Acinetobacter baumannii* | *tonB3* | NZ_CP00925 7 | IX87_RS18500 | WP_000525885.1 |
| *Acinetobacter baumannii* | *bauA* | NZ_CP00925 7 | IX87_RS08450 | WP_001073028.1 |
| *Acinetobacter baumannii* | *pfeA* | NZ_CP00925 7 | IX87_RS00590 | WP_000044170.1 |
| *Acinetobacter baumannii* | *piuC* | NZ_CP00925 7 | IX87_RS16645 | WP_085941346.1 |
| *Acinetobacter baumannii* | *feoB* | NZ_CP00925 7 | IX87_RS15455 | WP_001278226.1 |
| *Acinetobacter baumannii* | *feoA* | NZ_CP00925 7 | IX87_RS15450 | WP_001991212.1 |
| *Pseudomonas aeruginosa* | *pvdS* | NC_002516 | PA2426 | NP_251116.1 |
| *Pseudomonas aeruginosa* | *piuA* | NC_002516 | PA4514 | NP_253204.1 |
| *Pseudomonas aeruginosa* | *piuC* | NC_002516 | PA4515 | NP_253205.1 |
| *Pseudomonas aeruginosa* | *pirA* | NC_002516 | PA0931 | NP_249622.1 |
| *Pseudomonas aeruginosa* | *exbB 1* | NC_002516 | PA0198 | NP_248889.1 |
| *Pseudomonas aeruginosa* | *exbB 2* | NC_002516 | PA0693 | NP_249384.1 |
| *Pseudomonas aeruginosa* | *exbD 1* | NC_002516 | PA0199 | NP_248890.1 |
| *Pseudomonas aeruginosa* | *exbD 2* | NC_002516 | PA0694 | NP_249385.1 |
| *Pseudomonas aeruginosa* | *tonB1* | NC_002516 | PA5531 | NP_248888.1 |
| *Pseudomonas aeruginosa* | *tonB2* | NC_002516 | PA0197 | NP_248888.1 |
| *Pseudomonas aeruginosa* | *tonB3* | NC_002516 | PA0406 | NP_249097.1 |
| *Stenotrophomonas maltophilia* | *piuA* | NZ_CP00883 8 | DP16_RS16675 | WP_024958210.1 |
| *Stenotrophomonas maltophilia* | *piuC* | NZ_CP00883 8 | DP16_RS16685 | WP_012510312.1 |
| *Stenotrophomonas maltophilia* | *pirA* | NZ_CP00883 8 | DP16_RS15000 | WP_038645752.1 |
| *Stenotrophomonas maltophilia* | *exbB 1* | NZ_CP00883 8 | DP16_RS04645 | WP_005411738.1 |
| *Stenotrophomonas maltophilia* | *exbB 2* | NZ_CP00883 8 | DP16_RS09610 | WP_024956501.1 |
| *Stenotrophomonas maltophilia* | *exbD 1* | NZ_CP00883 8 | DP16_RS00790 | WP_024957123.1 |
| *Stenotrophomonas maltophilia* | *exbD 2* | NZ_CP00883 8 | DP16_RS09615 | WP_005409874.1 |
| *Stenotrophomonas maltophilia* | *tonB1* | NZ_CP00883 8 | DP16_RS21750 | WP_005410719.1 |
| *Stenotrophomonas maltophilia* | *tonB2* | NZ_CP00883 8 | DP16_RS04640 | WP_005411737.1 |
